# Use of micro‐CT to determine tracheobronchial airway geometries in three strains of mice used in inhalation toxicology as disease models

**DOI:** 10.1002/ar.24596

**Published:** 2021-03-06

**Authors:** Michael J. Oldham, Francesco Lucci, Clement Foong, Demetrius Yeo, Bahman Asgharian, Steve Cockram, Stephen Luke, Joanne Chua, Julia Hoeng, Manual C. Peitsch, Arkadiusz K. Kuczaj

**Affiliations:** ^1^ Altria Client Services LLC Richmond Virginia USA; ^2^ Oldham Associates LLC Manakin Sabot Virginia USA; ^3^ Philip Morris International Research and Development Neuchâtel Switzerland; ^4^ Philip Morris International Research Laboratories Pte. Ltd., Science Park II Singapore Singapore; ^5^ Applied Research Associates, Inc. Raleigh NC USA; ^6^ Synopsys Northern Europe Ltd., Bradninch Hall Exeter UK; ^7^ Multiscale Modeling and Simulation, Department of Applied Mathematics University of Twente Enschede The Netherlands

## Abstract

Aerosol dosimetry estimates for mouse strains used as models for human disease are not available, primarily because of the lack of tracheobronchial airway morphometry data. By using micro‐CT scans of in‐situ prepared lung casts, tracheobronchial airway morphometry for four strains of mice were obtained: Balb/c, AJ, C57BL/6, and Apoe^−/−^. The automated tracheobronchial airway morphometry algorithms for airway length and diameter were successfully verified against previously published manual and automated tracheobronchial airway morphometry data derived from two identical in‐situ Balb/c mouse lung casts. There was also excellent agreement in tracheobronchial airway length and diameter between the automated and manual airway data for the AJ, C57BL/6, and Apoe^−/−^ mice. Differences in branch angle measurements were partially due to the differences in definition between the automated algorithms and manual morphometry techniques. Unlike the manual airway morphometry techniques, the automated algorithms were able to provide a value for inclination to gravity for each airway. Inclusion of an inclination to gravity angle for each airway along with airway length, diameter, and branch angle make the current automated tracheobronchial airway data suitable for use in dosimetry programs that can provide dosimetry estimates for inhaled material. The significant differences in upper tracheobronchial airways between Balb/c mice and between C57BL/6 and Apoe^−/−^ mice highlight the need for mouse strain‐specific aerosol dosimetry estimates.

## 1 INTRODUCTION

Laboratory mice, including transgenic mice, are used in a variety of in vitro and in vivo pulmonary toxicology models of human disease. For the laboratory mice used in in vivo inhalation toxicology studies, it is essential that the respiratory tract anatomy is accurately described in order to derive estimates of regional dosimetry (nasal, tracheobronchial, and alveolar) of toxins, toxicants, and therapeutics. The estimates of regional dosimetry are obtained through a combination of anatomical, physiological, and aerosol physics considerations in aerosol dosimetry programs, such as the Multiple‐Path Particle Dosimetry (MPPD) model (Asgharian et al., 2014; RIVM, 2002), ICRP (ICRP, 1994), NCRP (NCRP, 1997), or computational fluid dynamics techniques (Frederix et al., 2018; Zhang, Kleinstreuer, Donohue, & Kim, 2005). These dosimetry estimates are also critical when there are no biomarkers of exposure that can be reliably measured from animals in inhalation studies. For example, detailed knowledge of the nasal anatomy of the Sprague–Dawley rat helped explain the lesion loci for formaldehyde exposure (Kimbell, Gross, Richardson, Conolly, & Morgan, 1997), and detailed knowledge of the upper tracheobronchial anatomy of B6C3F_1_ and Balb/c mice helped explain the 12‐fold difference in response to the same concentration of methacholine (Moss & Oldham, 2011).

Aerosol dosimetry programs and computation fluid dynamic techniques use airway length, diameter, branch angle, and inclination to gravity to provide regional dosimetry estimates throughout the respiratory tract. Depending on the aerosol being modeled, typically three particle deposition mechanisms (diffusion, impaction, and sedimentation) are used to calculate particle deposition within the airways. The inclination to gravity of an airway is used for calculating particle deposition that occurs from sedimentation for micron‐sized particles. Due to the physiological difference between humans (bipedal) and rodents (four legged), different initial assumptions for inclination to gravity are required in the tracheobronchial region that begins with the trachea, parallel to force of gravity for humans and 90° to gravity for rodents.

Advancements in imaging, specifically high‐resolution micro‐computed tomography (micro‐CT), have enabled us to obtain laboratory rodent respiratory tract anatomical data from infused in vivo specimens and lung casts. Micro‐CT has been used to obtain detailed tracheobronchial anatomical data in rats for some time (Lee, Fanucchi, Plopper, Fung, & Wexler, 2008). However, because of the size difference between rats and mice (approximately, a factor of 10), it is only recently that detailed tracheobronchial airway measurements based on micro‐CT scans of in‐situ prepared mouse lung casts have been published (Islam et al., 2017). Islam et al. (2017) provided detailed tracheobronchial airway measurements for the Balb/c mouse, which has been used as a sensitization model in inhalation studies of air pollution, air‐borne allergens, and toxicants (Barrett et al., 2011; Barrett, Henson, Seilkop, McDonald, & Reed, 2006; Yoshizaki et al., 2017). Thiesse et al. (2005, 2010) used limited upper tracheobronchial anatomical data of the C57BL/6 mouse to conclude that it is unique compared to previously reported mouse tracheobronchial anatomy (Oldham & Phalen, 2002; Oldham, Phalen, Schum, & Daniels, 1994). Ford et al. (2007) studied breath waveforms and the range of tracheal and main bronchial dimensions during breathing in C57BL/6 mice by using micro‐CT techniques. Estimates of regional dosimetry obtained by using dosimetry programs only exist for CF_1_, Balb/c, and B6C3F_1_ mice (Asgharian et al., 2014; Madl, Hofmann, Oldham, & Asgharian, 2010; Oldham et al., 1994; Oldham & Phalen, 2002; Oldham & Robinson, 2007; Raabe, Al‐Bayati, Teague, & Rasolt, 1988). Recently, Bauer, Krueger, Lamm, Glenny, and Beichel (2020) developed an approach for generating high‐resolution lung anatomical data from serial block‐face cryomicrotome images. Their analysis resulted in 34 mouse models from four different mouse strains (B6C3F_1_, BALB/c, C57BL/6, and CD‐1), including both sexes, and the models were publicly shared.

The present study focused on three strains of mice (AJ, C57BL/6, and Apoe^−/−^) that are used as models of human disease in inhalation studies. The AJ mouse has been used as a model of lung cancer in inhalation studies (Witchi, Witschi, 1998; Witschi, 2005; Glauert et al., 2013). The C57BL/6 mouse is moderately deficient in the serine protease inhibitor Serpina1 (more pronounced in female mice) and has been used as a model of chronic obstructive pulmonary disease in inhalation studies (Ansari et al., 2016; Bartalesi et al., 2005; Lee et al., 2019). The Apoe^−/−^ mouse is an apolipoprotein E‐deficient transgenic strain used as a model for atherogenesis (Veniant, Withycombe, & Young, 2001; Lippman et al., Lippmann, Gordon, & Chen, 2005), especially in inhalation studies on smoking‐related atherosclerosis (Boue et al., 2012; Phillips et al., 2019). The present study used micro‐CT images of in‐situ prepared lung casts to derive tracheobronchial airway measurements (airway length, diameter, branch angle, and inclination to gravity) from AJ, C57BL/6, and ApoE^−/−^ mice. To verify the micro‐CT scans and techniques used to derive the tracheobronchial airway measurements, our micro‐CT‐derived tracheobronchial airway measurements were compared with previously published data (Islam et al., 2017) and manual morphometry measurements. The goal of this study was to provide tracheobronchial airway morphometry data that will be suitable for use in aerosol dosimetry programs.

## 2 MATERIALS AND METHODS

### 2.1 In‐situ lung casts

All in‐situ mouse lung casts used in this study were made by using the saline replacement method originally described by Phalen, Yeh, Raabe, and Velasquez (1973) and subsequently refined by others (Madl et al., 2010; Oldham et al., 1994; Oldham & Phalen, 2002; Oldham & Robinson, 2007). Briefly, mice were injected with a lethal dose of sodium pentobarbital (120 mg/kg). After confirming the absence of any blink and pinch reflex, a tracheostomy was performed with the trachea nicked (opened, not severed) between the third and fifth cartilaginous ring. Polyethylene tubing covering a luer lock blunt end cannula was inserted and tied in the trachea between the seventh and 10th cartilaginous ring; then, the abdominal cavity was opened, and pneumothorax was performed by puncturing the diaphragm. The lungs were ventilated with 100% CO_2_ (at ≤25 cm H_2_O) to remove nitrogen, which does not dissolve in degassed saline. After a minimum of 10 lung inflation/deflation cycles (bubbles coming out of the tubing submerged in a column of water), degassed saline was injected into the lungs with a target volume of 0.35% of body mass. The CO_2_ readily dissolves in the saline which is then displaced (saline diffuses out of the pleura) by the injected casting material. This prevents formation of gas bubbles that could result in an incomplete cast. Casting material was put into a 1 or 3 cc syringe and injected into the lung at 0.15–0.2 ml/min using a syringe pump (mechanical for Balb/c and AJ mouse casts; electronic for C57BL/6 and ApoE^−/−^ mouse casts) until the target amount of casting material, 0.35% of body mass or casting material was seen in alveoli in the diaphragmatic lobes through the diaphragm. After the target amount of casting material was injected or casting material was seen in alveoli in the diaphragmatic lobes, injections were stopped and the syringe and cannula were removed. After curing for approximately 24 hr, the lungs were removed and placed into 5–7 M NaOH solution to dissolve the tissue. Subsequently, the NaOH was neutralized with 2% vinegar; the lung cast washed and stored in isopropyl alcohol until manual morphometry measurements and/or micro‐CT images were acquired. The pressure level used for ventilating the lungs with CO_2_ and instilling degassed saline was kept at <25 cm H_2_O using a “T” with one end of the tubing submerged 24 cm in a graduated cylinder filled with water. The pressure level for injecting casting material was kept at <25 cm H_2_O, using an injection speed no faster than 0.15–0.2 ml/min so that pressures were consistent with the recent requirements of the American Thoracic Society and European Respiratory Society for quantitative assessment of lung structure (Hsia, Hyde, Ochs, & Weibel, 2010). The casting material used for the Balb/c and AJ in‐situ mouse lung casts was Silastic E (Dow, Midland, MI). Since Silastic E was replaced with XIAMETER RTV‐4230‐E (Dow, Midland, MI), it was used for the C57BL/6 and Apoe^−/−^ in‐situ mouse lung casts. Both materials are white platinum cure‐based room temperature vulcanizing silicone rubbers with a working time of 2 hr, a viscosity of 55,000 mPa.s at 25°C, specific gravity of 1.14, tensile strength of 5.5 MPa and a linear shrinkage of 0.1% after 7 days. The procedures for making the Balb/c and AJ in‐situ mouse lung casts were reviewed and approved by the institutional animal care and use committee of University of California (Irvine, CA). The procedures for making the C57BL6 and Apoe^−/−^ in‐situ mouse lung casts were reviewed and approved by the animal care and use committee of Philip Morris International Research Laboratories (PMIRL; Singapore). The PMIRL in Singapore are accredited by the American Association for the Accreditation of Laboratory Animal Care and licensed by the Agri‐Food & Veterinary Authority of Singapore. Two Balb/c in‐situ lung casts made in 2001 (Listed as Casts #2 and 3 in Islam et al., 2017) are listed as Casts #1 and #2 and three AJ in‐situ lung casts made in 2005 listed as Casts #3–5 were used in this study (Table 1). The five C57BL/6 and four Apoe^−/−^ in‐situ lung casts used in this study were made in 2018 (Table 1).

**TABLE 1:** Characteristics of the mice

Segmented cast #	Mouse strain	Sex	Body mass (g)	Age (days)	Length (rump to snout; cm)	Circumference at (cm)	
						Forearms	Xyphoid
1	Balb/c^a^	M	24.4	67	9.5	7.3	7.6
2	Balb/c^a^	M	25.9	67	9.7	7.3	7.8
	AJ^b^	M	24.9	79	9.0	6.8	7.2
3	AJ	M	25.2	79	9.2	6.7	7.2
	AJ^b^	M	24.5	79	9.4	6.8	7.2
4	C57BL/6	M	24.4	84–98	9.3	7.6	NA
5	C57BL/6	M	27.5	91–105	9.8	6.0	NA
6	C57BL/6	F	30.6	105–119	9.7	5.3	6.0
7	C57BL/6	F	23.8	112–126	10.1	7.1	7.5
8	C57BL/6	F	21.7	112–126	9.1	5.3	6.7
9	Apoe^−/−^	F	25.0	126–140	10.2	7.6	7.1
10	Apoe^−/−^	F	23.4	126–140	9.8	7.1	7.0
11	Apoe^−/−^	F	25.5	126–140	10.3	7.2	6.8
12	Apoe^−/−^	F	23.6	280–294	10.0	6.6	7.3

Abbreviation: NA, not available.

^a^Balb/c lung casts listed as 2 and 3 in Islam et al., 2017.

^b^ Casts could not be segmented because of casting artefacts of air within the silicone rubber.

### 2.2 Manual airway morphometry measurements

**FIGURE 1:**
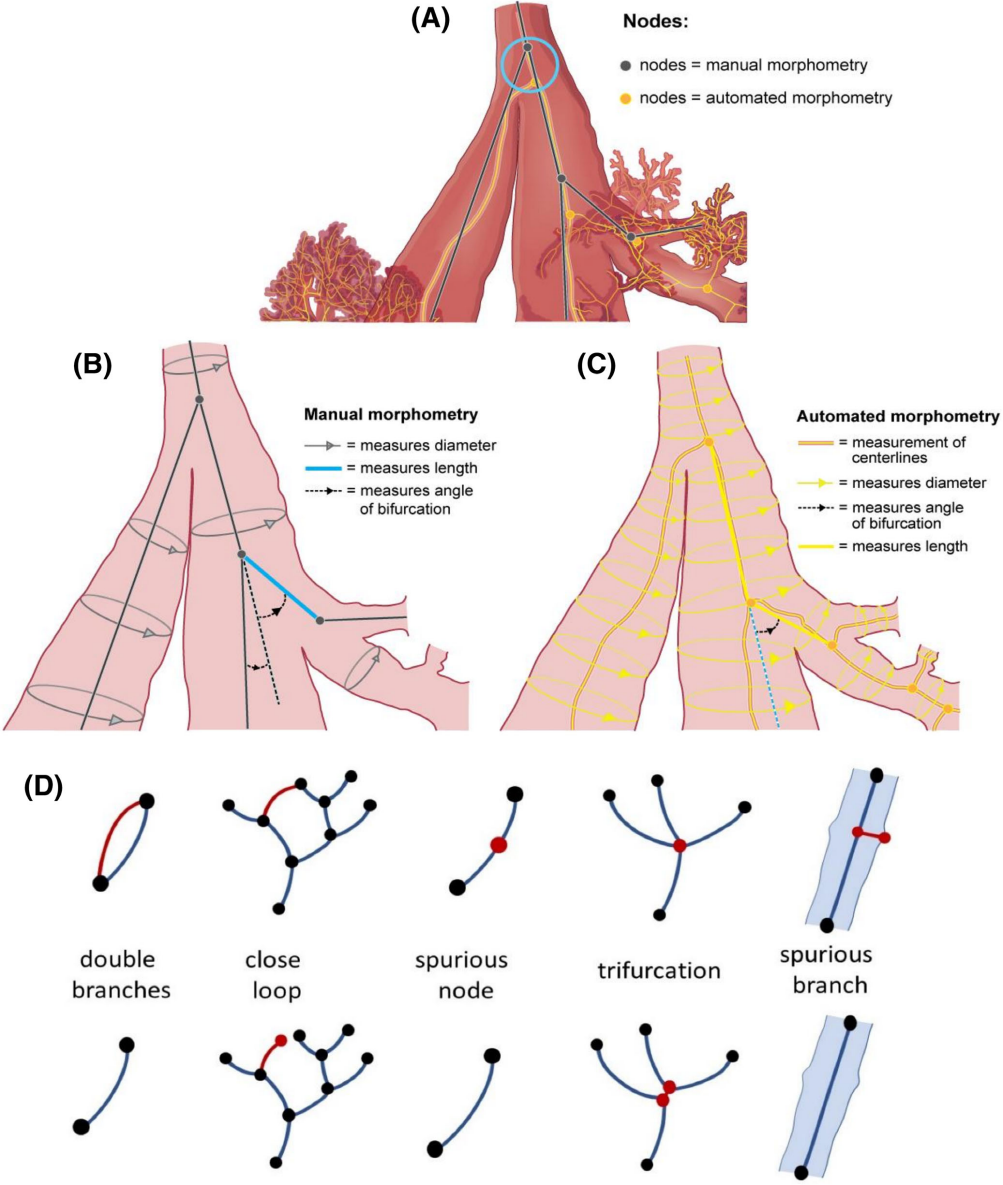
Airway model defining airway nodes, length, diameter, and branch angle both in the manual and automatic algorithms. Different node placement in the two procedures in a real geometry A (top). Schematic of manual measurements B (middle left). Schematic of automatic centerline generation and measurements C (middle right). Centerlines exceptions that are treated manually or with the assistance of automated scripts D (bottom) with top row a schematic of the exception and bottom row a schematic of the resolution

Manual morphometry measurements derived from the Balb/c and AJ in‐situ lung casts have been reported previously (Islam et al., 2017; Moss & Oldham, 2008; Oldham & Phalen, 2002; Oldham & Robinson, 2007). Manual morphometry measurements of the C57BL/6 and Apoe^−/−^ in‐situ lung casts were performed in accordance with the previous manual morphometry measurements by the same morphometrist and by using the same equipment. A binary number scheme (Phalen, Yeh, Schum, & Raabe, 1978) was used to identify airways, and airway length, diameter, and branch angle were measured (Figure 1) for every airway in the first six airway generations (trachea = airway generation 1) by using a 10x magnifying lens. The airways' inclination to gravity was also measured when possible, assuming that the trachea was inclined at 90° to gravity. The assumption of 90° to gravity was made so the inclination to gravity measurements would be consistent with how mice are exposed in whole body and nose‐only inhalation toxicology studies. Airway lengths and diameters were measured to the nearest 0.05 mm. Branch and inclination to gravity angles were measured to the nearest 1°. The measurements from the Balb/c in‐situ lung casts were presented as summary statistics in previous publications, with the detailed measurements presented by Islam et al. (2017). The measurements from the AJ in‐situ lung casts have only been presented as summary statistics and never reported to the level of detail presented here (Data S1). The measurement precision combined with the stability of the material used for making the Balb/c in‐situ lung casts in 2001 was assessed by Islam et al. (2017). The authors (Islam et al., 2017) used remeasurements by the original morphometrist to determine that the absolute difference (mean ± *SD*) of the combined measurement precision and stability of the cast material (14 years old at time of remeasurement) for the in‐situ Balb/c lung casts was 0.032 (±0.05) mm for airway diameter, 0.067 (±0.12) mm for airway length, and 6.6 (±12) degrees for airway branch angle. The same precision is assumed for the measurements derived from the AJ in‐situ lung casts, because these casts were made from the same material and stored identically (suspended in isopropyl alcohol) and are of similar age. The same precision is also assumed as the worst case for the measurements derived from the C57BL/6 and Apoe^−/−^ in‐situ lung casts, because the airway measurements were made by the same morphometrist using the same methods and magnifying lens.

### 2.3 Micro‐CT‐derived morphometry measurements

**FIGURE 2:**
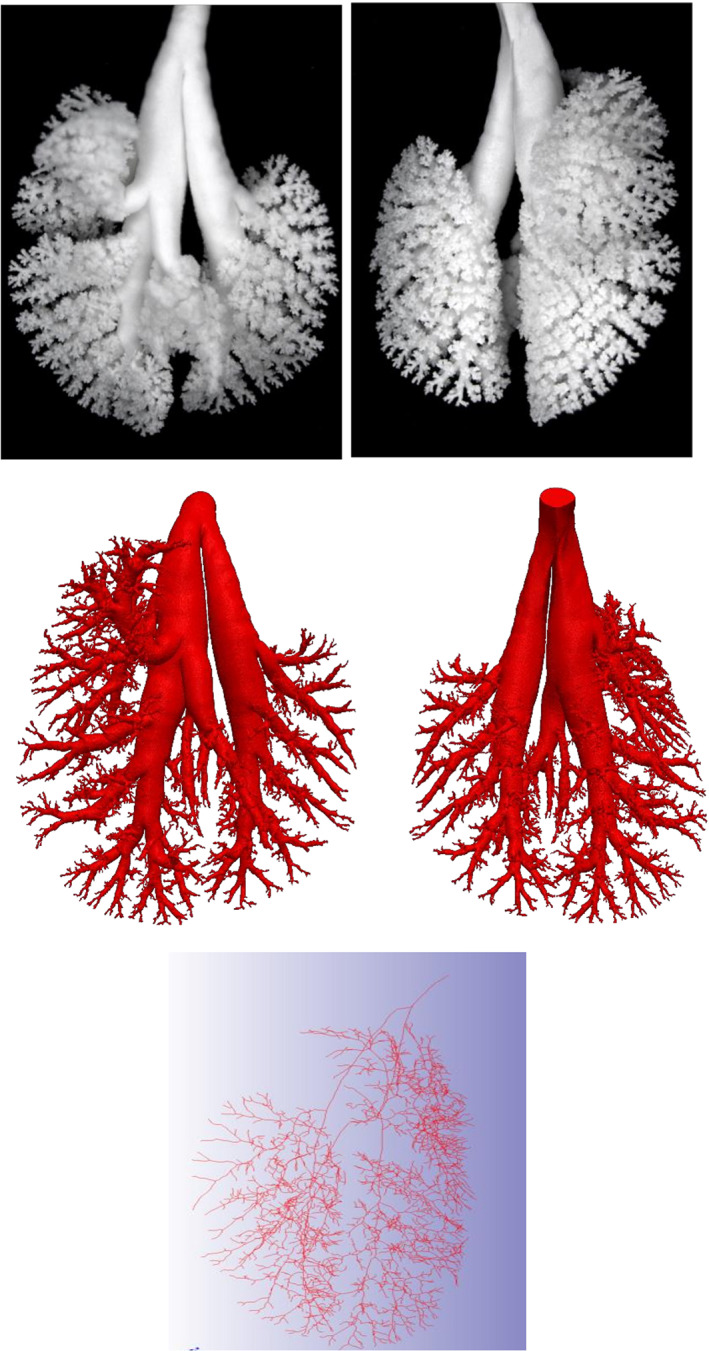
Apoe^−/−^ in‐situ‐prepared lung cast (Cast 11) in the top panel. Middle panel shows 3D reconstruction of the same Apoe^−/−^ in‐situ‐prepared lung cast, while the bottom panel shows the skeletonization. In the top and middle panels, ventral view on the left and dorsal view on the right

**FIGURE A1:**
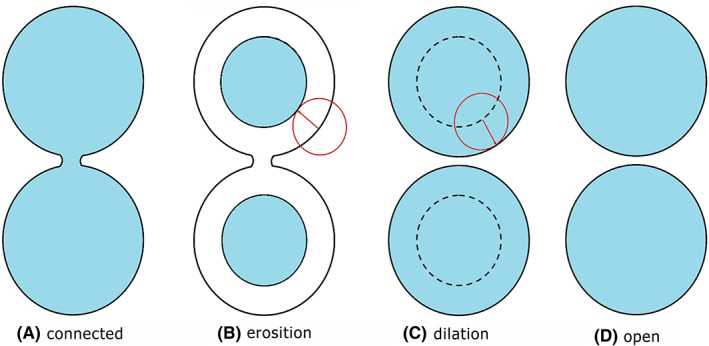
Sketch, illustrating the open operation used in image processing to separate weakly connected segmented regions. The open operation is the combination of an erosion followed by a dilation of the same operator width highlighted by a red circle

Micro‐CT images of the Balb/c, AJ, C57BL/6, and Apoe^−/−^ in‐situ lung casts were acquired by using a μCT 100 scanner (SCANCO Medical AG, Switzerland) at a 6.6 μm voxel resolution. By using existing methods (Young et al., 2008), as implemented in the commercial software package Simpleware ScanIP (Version N‐2018.03; Synopsys, Inc., Mountain View, USA), scans from 12 in‐situ mouse lung casts were segmented and reconstructed into a 3D model of each individual lung cast (Figure 2). The final 3D model for each lung cast was generated using an image analysis protocol (Appendix A) by applying, among others, a threshold (Rogowska, 2000) and the mathematical morphological opening operation (Haralick & Shapiro, 1985; Sapiro, Kimmel, Shaked, Kimia, & Bruckstein, 1993) which was necessary to remove artificial points of contact between branches caused by the lack of resolution of the segmentation process (see Figure A1). These points of contact would create connections in the 3D model resulting in closed loops in the branching tree compromising the subsequent skeletonization process. The segmentation parameters were determined by the combined quality of the in‐situ cast and micro‐CT images, some reference values used in the present dataset are in the image analysis protocol (Appendix A). Because of a memory limit, during segmentation, the images were resampled with a factor of 2, and an opening operation was then performed by using a structuring element with typically 2 pixels of size, which is equivalent to an opening bandwidth of four voxels. Consequently, the accuracy of the present workflow can be estimated as twice the bandwidth or about 50 μm. Any detail smaller than this dimension is lost causing the 3D model and the centerline to end whenever a restriction in the branching of this dimension is encountered. A centerline network of each processed lung cast was automatically created by using the centerline tool available in Simpleware ScanIP which shrinks the 3D model of each airway to its medial axis and fitting a spline to this axis (Figure 2). At the intersection of two centerlines, a bifurcation node was created (Figure 1). After its creation, the centerline network was visually inspected to remove possible spurious branches that might have been created by the presence of annular ligaments or by other artifacts caused by the segmentation process (Figure 1d). Taking advantage of the scripting options of Simpleware ScanIP, some scripts were written to automatically detect possible centerline network exceptions like closed loops, trifurcations, and spurious nodes, which were subsequently manually resolved (Figure 1d). A final detailed visual inspection was performed to remove further anomalies that were not captured by the first visual inspection. Finally, the centerline network was automatically measured for extracting airway morphometry characteristics: airway length and diameter (mm), branch angle (degrees), and inclination to gravity angle (degrees). In addition, the total number of airways in each generation was counted.

**FIGURE 3:**
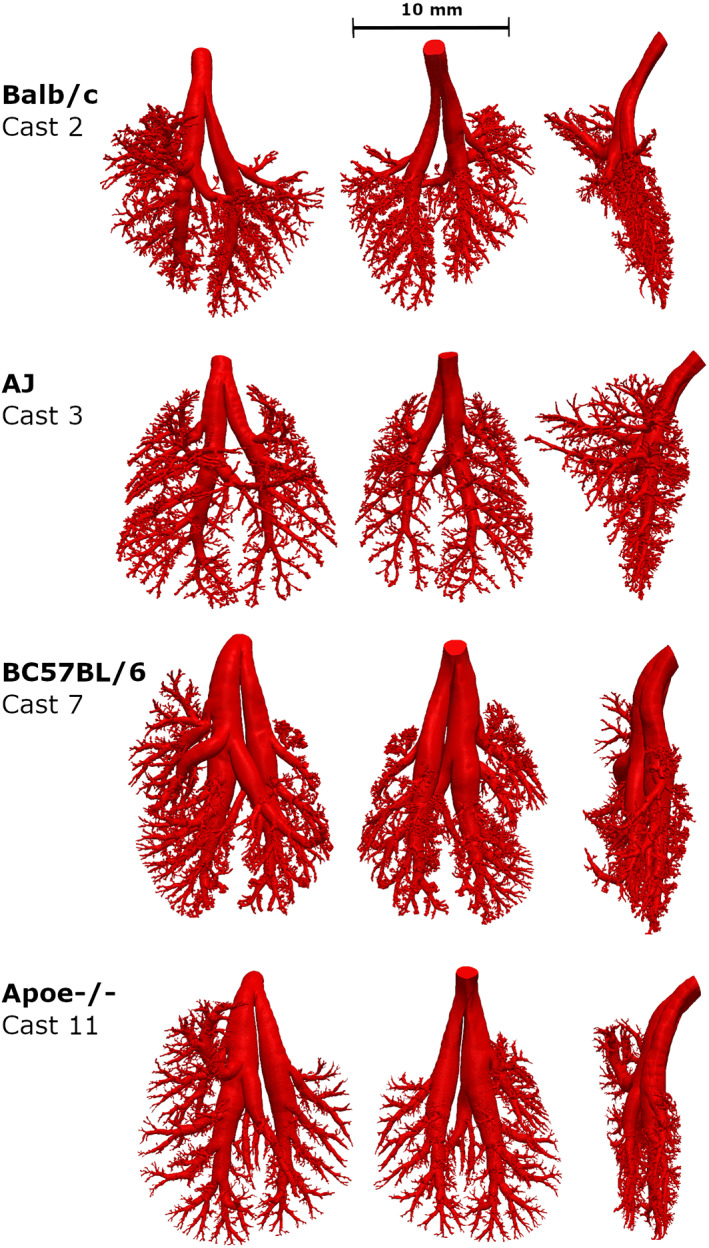
Comparison of ventral (left), dorsal (center), and side (right) views of a reconstructed 3D lung model for each mouse strain

Airway length was measured as the linear distance between two bifurcations nodes (Figure 1c). Note that to optimize the ratio between accuracy and required data, Micro‐CT images were generated starting from the base of the trachea to limit the scanned volume. This resulted in a very short trachea in the 3D reconstructed models (Figure 3), consequently automatic measurements of the trachea are not reported. Airway diameter was based on the hydraulic diameter (average) along the centerline of each branch (Figure 1c). The hydraulic diameter was computed from cross‐sections perpendicular to the branch centerlines. If a cross‐section was crossing the centerline of another branch it was discharged and it was not contributing to the branch diameter average. When the procedure failed to measure a diameter, a flag was attributed to the branch, and its diameter was set to zero. The value of the parent was then used to compute the generation averages. Failure of hydraulic diameter measurement can happen when the branch length is shorter than the diameter or the branch is highly bent. The branch angle was computed from the angle between the parent and daughter airway vectors defined by the bifurcation nodes (Figure 1c). To compute the angle to gravity for each airway, we assumed gravity to be perpendicular to the trachea and laying on the plane defined by the trachea and the average vector of the two main bronchi. The automated airway morphometry algorithm assigned the binary airway identification number on the basis of a comparison of daughter airway diameters in the same manner as the manual morphometry techniques following the convention by Raabe, Yeh, Schum, and Phalen (1976).

### 2.4 Comparison of morphometry measurements

The binary number scheme identified each airway with a unique identification number consisting of 1 and 2 s. The trachea was defined as 1. The label for each daughter airway incorporated the parent label but added a 1 or 2 depending on the comparison of daughter airway diameters. For example, because the right main bronchus is larger (major daughter) than the left main bronchus, its identification number is 11, while the identification number for the left main bronchus is 12 (minor daughter). Using the binary number scheme, an airway generation is defined as the group of all airways with the same number of 1 and 2 s. For Casts 1 and 2, corresponding branches were compared between the micro‐CT‐derived morphometry measurements from this study and the manual and micro‐CT‐derived morphometry measurements reported by Islam et al. (2017), when both datasets had airway morphometry data. Corresponding branches were also compared between the micro‐CT‐derived morphometry measurements from this study and the manual morphometry measurements for Casts 3–12, when both datasets had airway morphometry data.

Airway length, diameter, and branch angle (Figure 1) were compared by using a least‐squared solution (Numpy Python library; Oliphant, 2006, 2007), with the intercept forced through (0,0) to indicate goodness of fit. Pearson correlation coefficient, *r*, was computed by using the stats.pearsonr function of the SciPy library (Virtanen et al., 2020). As noted by Islam et al. (2017), there was occasionally what appeared as a trifurcation, where the parent airway split into three separate daughter airways. In manual morphometry, a trifurcation is split into two bifurcations, with an intermediate airway arbitrarily assigned an airway length of 0.1 mm and a branch angle of 0° along with the measured diameter. There were two to four trifurcations in Casts 1 and 2, one in Cast 8, and two in Cast 10, with none in the first six airway generations of the other casts based on manual morphometry measurements. These trifurcations were manually adjusted for in the automated airway morphometry technique, and the diameter of the parent airway was used for the airway created.

Micro‐CT‐derived average generation data (airway values with an airway generation were averaged) for each mouse strain was compared for significant differences in airway length, diameter, branch angle, and inclination to gravity using ANOVA (SciPy Python library; Virtanen et al., 2020) with results reported for *p* < .05 and .01. Our criteria for a statistically significant difference was *p* < .05. To isolate differences between strains, paired *t* tests with Bonferroni correction were performed using statistical significance *p* < .01. Because the C57BL/6 and ApoE^−/−^ mice used in this study were a convenience sample (obtained from exiting ongoing studies) differences in animal sex and age were possible. The potential effect of mouse age and sex on average airway length and diameter was evaluated using paired *t* tests (SciPy Python library; Virtanen et al., 2020). If systematic differences were found with the paired *t* tests, ANOVA was used to take into account mouse, age, sex, body mass, and body length to assess which factors accounted for any observed differences.

## 3 RESULTS

**FIGURE 4:**
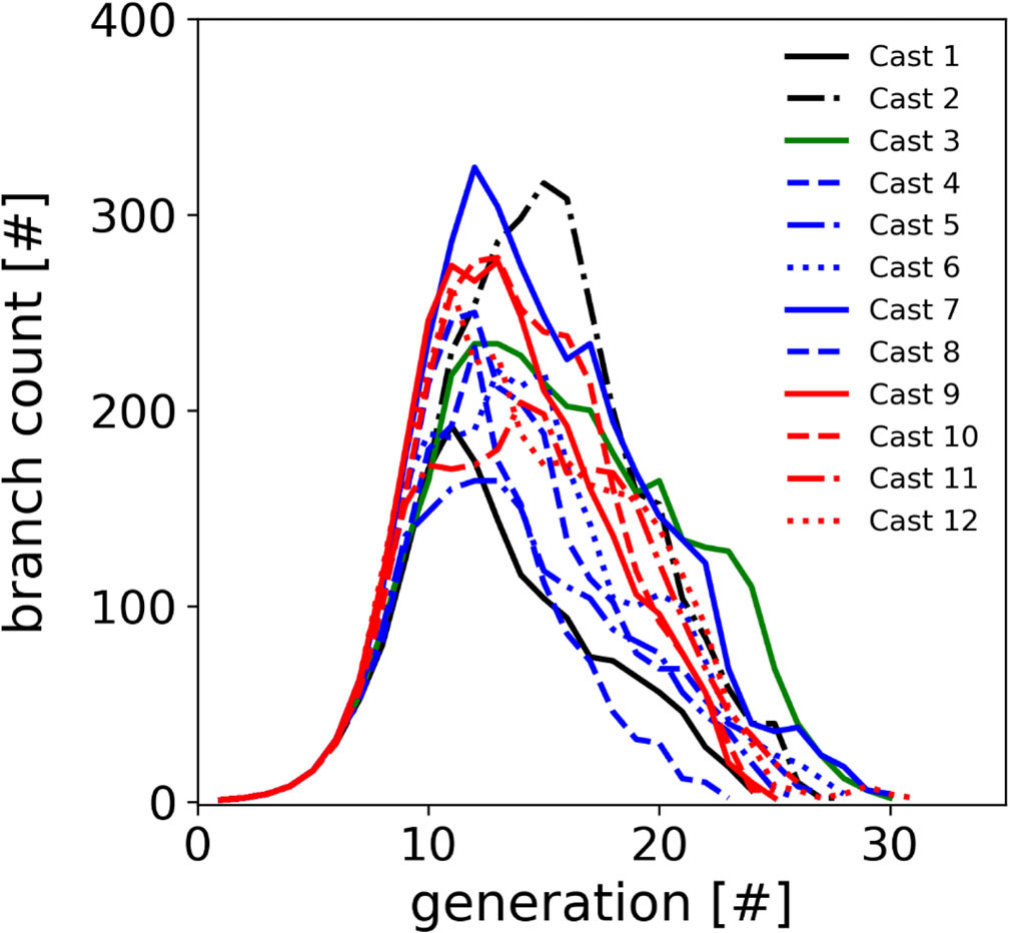
Number of tracheobronchial airways per generation for each cast using the automated micro‐CT techniques. Each mouse strain has a different color line (black = Balb/c; green = AJ; blue = C57BL/6; and red = ApoE^−/−^) with casts distinguished by line style

The representative 3D reconstructed models for each mouse strain (Figure 3) visually appear to show significant differences in the upper tracheobronchial airway morphology. For example, the Balb/c and AJ 3D mouse models appear to have smaller airway diameters than the C57BL/6 and Apoe^−/−^ 3D mouse models for the airways branching into the right apical and medial lobes. In Apoe^−/−^ mice, the airway branching into the right medial lobe makes an almost 180° turn, which is unique among the four strains of mice. The manual airway morphometry technique was able to measure all 63 airways in the first six airway generations for all but three casts (i.e., Casts 5, 7, and 9). In the manual airway morphometry method, Cast 5 was filled with too much casting material, which prevented manual airway morphometry measurement. In Cast 7, a terminal bronchiole was identified in airway generation 5, which meant that there would be two less sixth airway generation airways; in Cast 9, two airways in airway generation 6 were inadvertently omitted. The automated technique measured 61 of 63 airways in Casts 2, 5, 10, and 11 and missed the terminal bronchiole identified by manual morphometry in Cast 7. The number of airways in each tracheobronchial airway generation using the automated airway morphometry technique did not start to diverge between mouse strains until generation 6 (Figure 4) where terminal bronchioles started to be identified in the manual morphometry measurements for some casts (Data S1).

**FIGURE 5:**
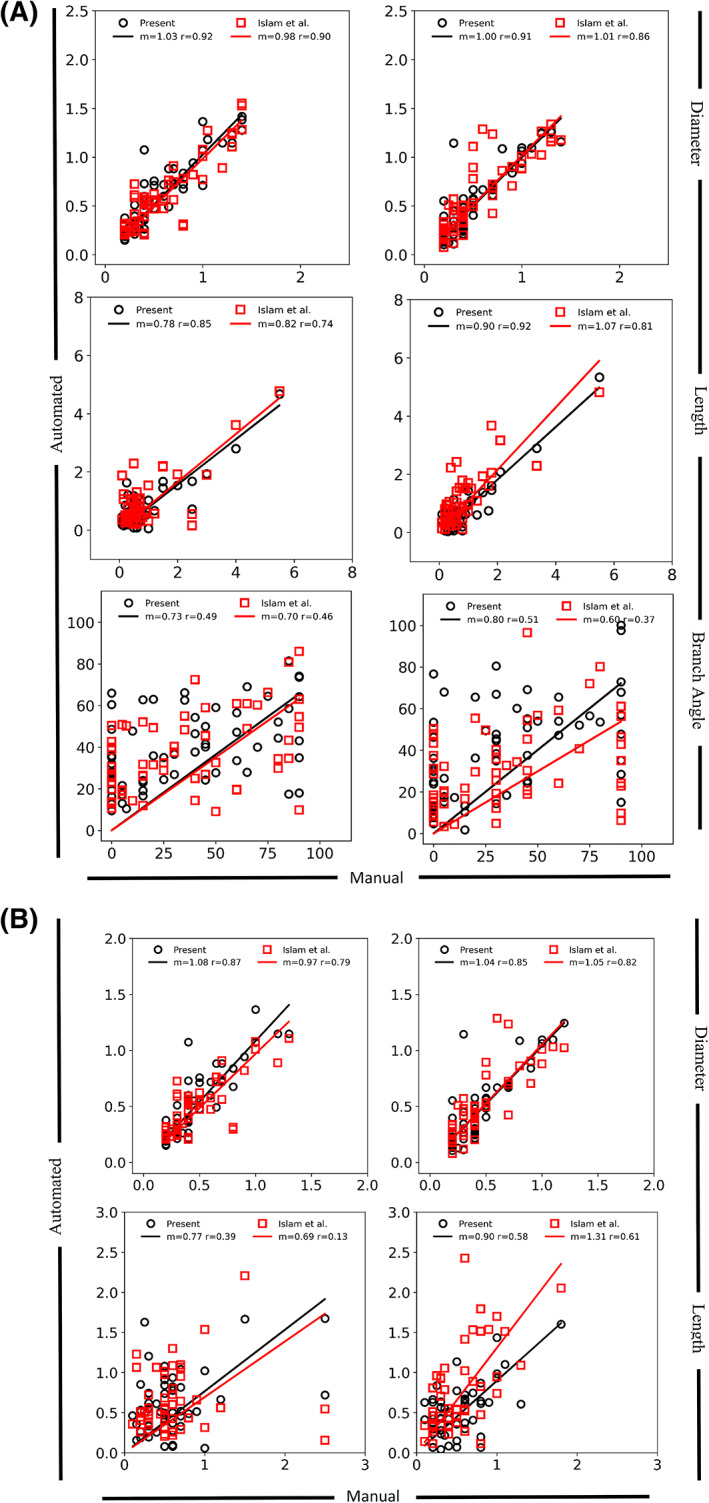
(A) Branch‐by‐branch comparison of airway diameter (top), length (middle), and branch angle (bottom) obtained from manual airway morphometry measurements of two in‐situ lung Casts, #1 and #2 (Balb/c), reported by Islam et al. (2017). Automated measurements of the present work (open circles) and those reported by Islam et al. (2017) (open boxes) for the first six tracheobronchial airway generations. (B) Branch‐by‐branch comparison of airway length obtained from manual airway morphometry measurements of two in‐situ lung Casts, #1 and #2 (Balb/c), reported by Islam et al. (2017). Automated measurements of the present work (open circles) and those reported by Islam et al. (2017) (open boxes) for three tracheobronchial airway generations (4, 5, and 6), which remove airway diameters of 1.5 mm or greater and lengths of 3 mm or greater

Branch‐by‐branch comparison of airway length and diameter between the manual and micro‐CT‐derived airway morphometry measurements published by Islam et al. (2017) and the micro‐CT‐derived airway morphometry measurements from the current study (Figure 5a) showed excellent agreement for the two Balb/c lung casts. For airway length, the automated airway morphometry data from the present study had a slightly better correlation to the manual airway morphometry data. For branch angles, the automated data from the current study and from Islam et al. (2017) both had similarly poor correlations to manual airway morphometry data. Removal of the longer length and larger diameter airways (airway generations 1, 2, and 3) from the branch‐by branch analysis (Figure 5b), results in lower correlation between the manual and micro‐CT‐derived airway morphometry measurements published by Islam et al. (2017) and the micro‐CT‐derived airway morphometry measurements from the current study. Except for airway length in Cast 1, the automated airway morphometry data from the present study had a slightly better correlation to the manual airway morphometry data (Figure 5b). Comparison of automated airway morphometry data from the current study with that of Islam et al. (2017) on an airway generation basis (airway values within an airway generation were averaged) for Casts 1 and 2 (Data S1) demonstrated minimal differences, with the only difference occurring for the airway branch angles of Cast 2.

**FIGURE 6:**
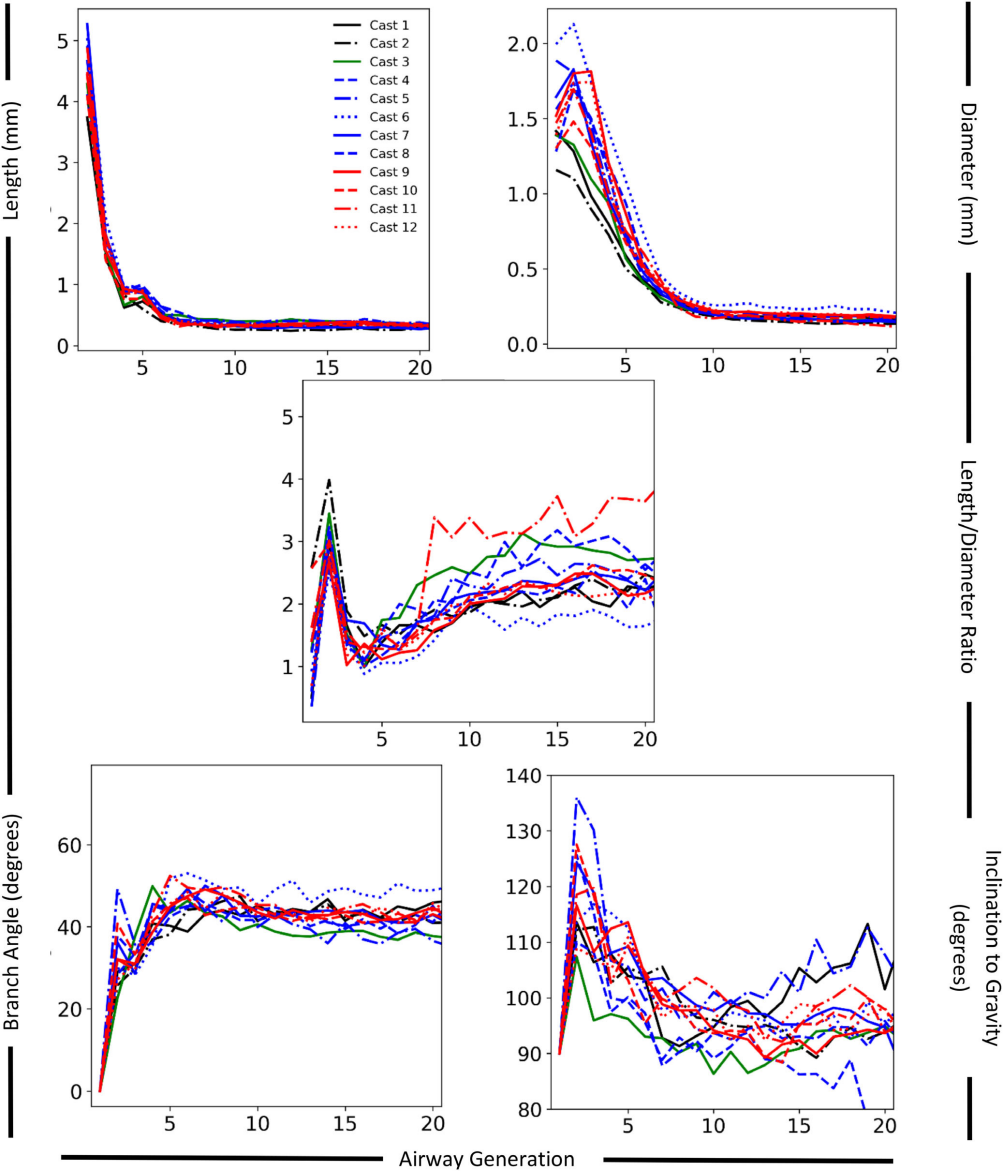
Average airway generation dimensions for airway length (top right), diameter (top right), length to diameter ratio (middle), branch angle (bottom left), and angle to gravity (bottom right) for all casts. Each mouse strain has a different color line (black = Balb/c; green = AJ; blue = C57BL/6; and red = ApoE^−/−^) with casts distinguished by line style (top left panel)

For Casts 3–12, the manual morphometry measurements (Data S1) for the first six airway generations were compared with the automated airway morphometry data on branch‐by‐branch (when both datasets had data) and airway generation (Figure 6, Table 2, and Figure S1) bases. There was good agreement in airway diameters and lengths for most casts, with the automated airway morphometry data consistently having larger airway diameter values (seven of 10 Casts) and smaller airway lengths (all casts). Except in Cast 5, there was poor correlation between the automated and manual branch angle measurements in the branch‐by‐branch comparison. In the average generation comparison, there was better agreement for all casts, with the automated airway morphometry data still having consistently smaller airway length values. Contrary to that in the airway‐by‐airway comparison, the automated airway morphometry data in the airway generation comparison had consistently larger branch angle values, and there was good correlation to manual morphometry measurements, except in one cast. The comparison of inclination to gravity angles showed significant differences because for airway generations 3–6 manual morphometry techniques were not able to measure the inclination to gravity angle for all airways as did the automated methods.

**TABLE 2:** Comparison of airway length, diameter, and branch angle on a branch‐by‐branch and an airway generation basis (all airways from the same generation are averaged) between manual and automated (this study) airway morphometry measurements for Casts 3–12

Cast number	Linear regression parameter for airways					
	Diameter		Length		Branch angle	
	Slope	*r*	Slope	*r*	Slope	*r*
Branch‐by‐branch comparison						
3	0.91	0.72	0.86	0.93	0.88	0.35
4	1.04	0.97	0.90	0.87	0.97	0.66
5	1.06	0.97	0.88	0.97	1.14	0.82
6	1.15	0.81	0.85	0.89	0.90	0.46
7	1.08	0.98	0.81	0.95	1.00	0.58
8	1.11	0.77	0.85	0.87	0.79	0.35
9	0.96	0.48	0.77	0.68	0.78	0.30
10	1.06	0.95	0.84	0.91	0.94	0.46
11	1.06	0.88	0.85	0.94	0.94	0.27
12	0.92	0.56	0.76	0.76	0.78	0.36
Airway generation comparison						
3	0.97	0.96	0.91	1.00	1.23	0.72
4	0.98	0.98	0.97	0.98	1.35	0.79
5	0.99	0.88	0.83	0.99	1.68	0.79
6	1.09	0.98	0.89	0.99	1.30	0.78
7	1.15	0.99	0.78	1.00	1.37	0.94
8	1.11	0.99	0.88	0.95	1.24	0.77
9	1.13	0.95	0.89	0.98	1.32	0.95
10	1.00	1.00	0.90	0.97	1.31	0.30
11	0.98	0.98	0.89	0.98	1.40	0.73
12	1.07	0.95	0.82	1.00	1.33	0.92

**FIGURE 7:**
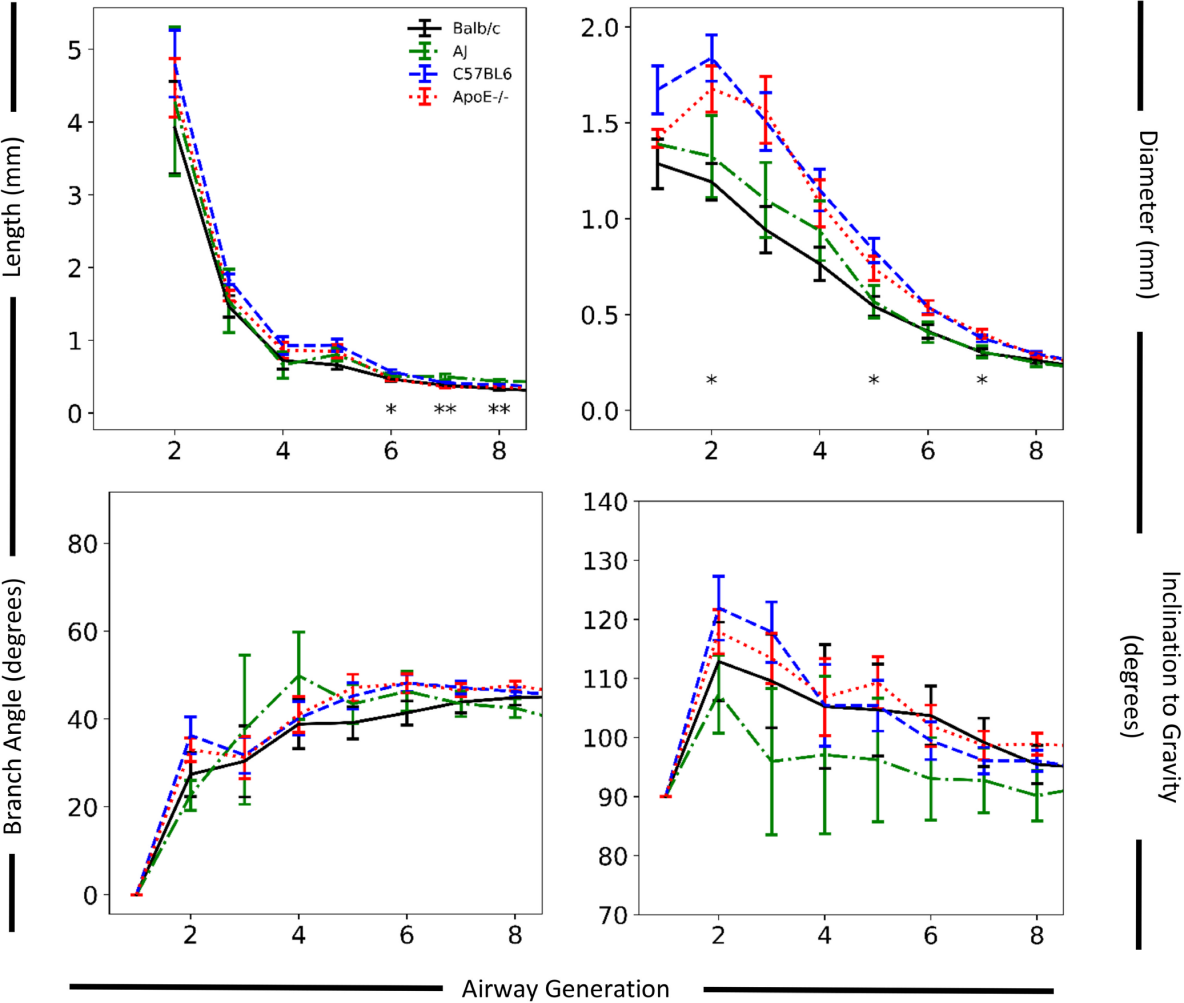
Average airway generation dimensions (±*SE*) for airway length (top right), diameter (top right), branch angle (bottom left), and angle to gravity (bottom right) for all strains. Asterisks indicate statistically significant differences with * and ** for *p* < 0.05 and *p* < 0.01, respectively. Each mouse strain is represented by a different line color as defined in the top left panel

Quantitative comparison of the automated airway morphometry on an average generation basis for airway length among the four mouse strains showed statistically significant differences (*p* < .05) in airway generations 6–8 (Figure 7 and Table S1). Similarly, quantitative comparison of the average generation data for airway diameter showed statistically significant differences (*p* < .05) among the four mouse strains in airway generations 2, 5, and 7 (Figure 7). Among the four strains of mice, there were no statistically significant differences in average generation data for airway branch angles or inclination to gravity (Figure 7). Pairwise *t* test comparisons showed one statistically significant difference (*p* < .01) in the first eight airway generations in the average generation data for airway length between Balb/c and AJ mice. No other statistically significant differences were found between average airway, diameter, branch angle, or inclination to gravity between Balb/c and AJ mice. Similarly, pairwise *t* test comparisons showed no statistically significant differences (*p* < .01) in the first eight airway generations between the average generation data for airway length, diameter, branch angle or inclination to gravity between C57BL/6 and ApoE^−/−^ mice. No statistically significant differences (*p* < .01) in average generation data for airway lengths in the first eight airway generations were found between Balb/c and C57BL/6 and ApoE^−/−^ mice. Pairwise comparison of the average generation data for airway diameter showed statistically significantly differences (*p* < .01) in airway generations 2–5 and 7 between Balb/c mice and C57BL/6 (Figure 7 and Figure S2). The average airway diameter was a minimum of 24% larger in C57BL/6 mice than Balb/c mice in the first seven generations, with airway generations 2, 3, and 4 being 54%, 60%, and 50% larger, respectively (Figures 7 and S2) confirming the visual appearance differences (Figure 3). Pairwise comparison of the average generation data for airway diameter showed statistically significantly differences (*p* < .01) in airway generations 2, 3, and 7 between Balb/c mice and ApoE^−/−^ mice (Figure 7 and Figure S2). The average airway diameter of ApoE^−/−^ mice for airway generations 2, 3, and 7 was 41%, 66%, and 32% larger than Balb/c mice, respectively, (Figure 7 and Figure S2).

## 4 DISCUSSION

Comparison of the automated airway morphometry data from this study with previously published manual and automated airway morphometry data (Islam et al., 2017) by using identical in‐situ Balb/c mouse lung casts showed excellent agreement in airway lengths and diameters despite some methodological differences between the two automated airway morphometry techniques. One difference is that, unlike in the study by Islam et al. (2017), there was no manual authentication of the binary airway identification numbers determined by the automated method in the present study by matching airway measurements of airway length and diameter to those reported by manual airway morphometry for all airways in the first six airway generations. Another major difference is that the airway dimensions by Islam et al. (2017) were extracted by substituting each airway with cylinders and matching the volume of the 3D cast to the volume of the cylinders. In the present study, airways were extracted starting by reducing the reconstructed 3D cast to its centerlines, which is a standard algorithm now available in the commercial software currently used. These differences account for some of the differences in airway length, diameter, and branch angle in the branch‐by‐branch comparisons between the two automated methods.

Differences between manual and automated definitions of airway length and diameter also contributed to the differences noted in our comparison. The manual airway morphometry definitions of airway length, diameter, and branch angle were developed for airway geometries in which the lengths and bifurcations are constructed from straight lines (Figure 1; also in Raabe et al., 1976) for use in aerosol dosimetry programs. The automated computer programs based on shrinkage of the 3D model are mathematically defined. Compared with the manual morphometry measurements, they have more resolution, but are less flexible in handling anomalies. For example, when the branch angle is relatively small and there is a significant difference in daughter airway diameters, which frequently happens in mouse upper tracheobronchial airways, the algorithm creates centerlines that will intersect (bifurcation node) at a position that is equidistant between the walls of each daughter branch, bending the centerline of the smaller diameter branch toward the centerline of the parent and larger diameter branch (Figure 1a) and crossing them almost perpendicularly (Figure 1c). The manual airway morphometry measurement centerlines intersect upward, projecting the branch along a straight line and thus crossing the parent branch with a narrower angle (Figure 1b). Similar discrepancies can be found when there is significant curvature along the airway length, which also frequently occurs in the mouse upper tracheobronchial airways, especially in airways entering the right apical (cranial) and medial lobes in C57BL/6 and Apoe^−/−^ mice (Figure 3). The difficulty in measurement of branch angles is consistent with that reported in the previous automated airway morphometry work, which noted that airways with short lengths were problematic (Islam et al., 2017). The automated airway morphometry techniques used in the current study provided the inclination to gravity angle for each airway, which was not possible in the manual airway morphometry technique. The inclusion of inclination to gravity angles, in addition to airway length, diameter, and branch angles, makes the current automated airway morphometry data directly usable in aerosol dosimetry programs like ICRP, NCRP, and MPPD (ICRP, 1994; NCRP, 1997; RIVM, 2002; Asgharian et al., 2014).

The current study has several limitations, including one that the C57BL/6 and Apoe^−/−^ mice used to obtain the in‐situ lung casts were a convenience sample. They were from a couple of different studies and, therefore, not as well matched for age, sex and body mass as the mice in some previous tracheobronchial airway morphometric studies (Islam et al., 2017; Oldham et al., 1994; Oldham & Phalen, 2002; Oldham & Robinson, 2007). Oldham and Phalen, (2002) did not find differences in tracheobronchial airway dimensions in the first six airway generations between groups of adult Balb/c mice 67 and 117 days old despite the older mice having 12% more body mass, 1.2 cm increase in rump to snout body length, a decrease in chest circumference at the axilla of 0.4 cm, but an increase in chest circumference at the Xyphoid of 0.1 cm). Comparing average airway length and diameter dimensions for the first six tracheobronchial generations derived from automated morphometry measurements using paired *t* tests between adult male and female C57BL/6 mice (Casts 4 vs. 7 and 5 vs. 6) found only one statistically significant difference (*p* ≤ .05) for average airway diameter in airway generation 5 between Casts 5 and 6 (Data S1). Suggesting that sex is not a factor in mouse airway dimensions. Although every attempt was made to fill each lung with an identical amount of casting material (0.35% of body mass), small errors can result in different numbers of airways being cast. When combined with another limitation of this study—that is, the lack of definitive criteria for identifying terminal bronchioles by using commercial segmentation/centerline network tools—the variability in number of airways per generation was consistent with those recently reported (Bauer et al., 2020) for Balb/c (30 airway generations) and C57BL/6 mice (26 airway generations). Another limitation in this study is that tracheal length was only derived from the manual morphometry measurements. With fixed computing resources, the cost and accuracy of the microCT scanning is a function of the scanned volume so we focused these resources on airways distal to the trachea. The commercial automated airway morphometry algorithms used in the current study had some difficulty in dealing with complex lung morphology, consistently underestimating airway length (both branch‐by‐branch and airway generation bases) and branch angles (branch‐by‐branch) in comparison to the manual airway technique; they still required visual inspection and, sometimes, intervention for excluding intrinsic errors. Automated airway morphometry techniques are significantly less time intensive than manual airway morphometry techniques, enabling analysis of more lungs, casts, or airways (e.g., as a function of growth, species, strains, and disease progression) that can provide better representation of average airway morphometry values and morphometric variability within a species or strain.

**FIGURE 8:**
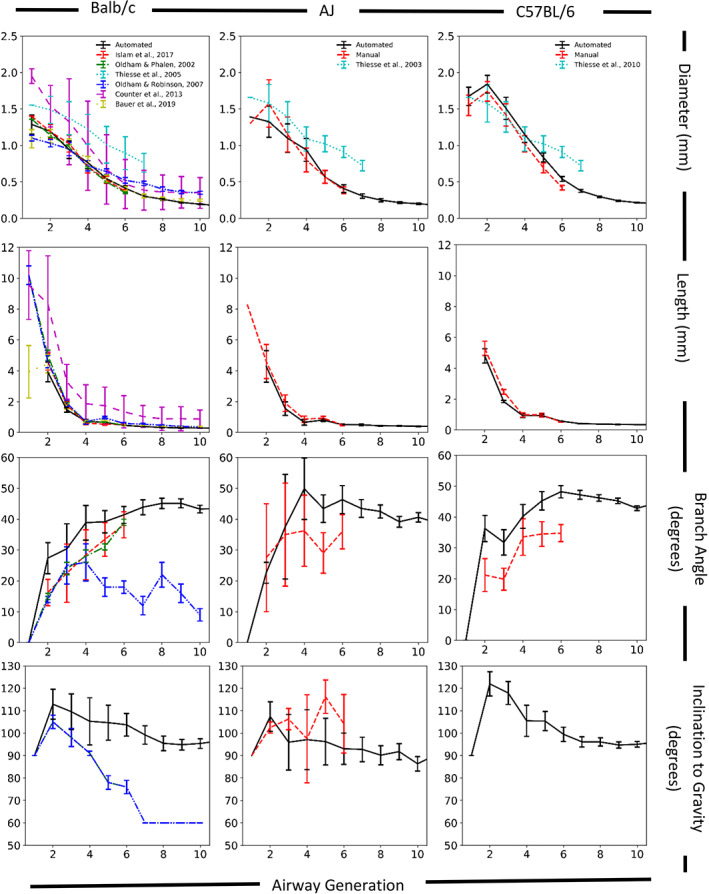
Comparison of automated average airways dimensions by mouse strain from the current study (automated = black lines; manual = red lines) with previously published data for Balb/c (left column), AJ (middle column), and C57BL/6 (right column) mice

The manual airway morphometry measurements for the two Balb/c in situ lung casts used in this study and by Islam et al. (2017) were also used in previous manual airway morphometry studies of Balb/c mice (Madl et al., 2010; Oldham & Phalen, 2002). They are also consistent with the manual measurements (airway‐by‐airway) of similarly prepared Balb/c mouse in situ lung casts (Oldham & Robinson, 2007) and previously reported micro‐CT‐derived average airway generation morphometry measurements in Balb/c mice (Bauer et al., 2020; Counter, Wang, Farncombe, & Labiris, 2013; Figure 8). Airway lengths and diameters reported by Counter et al. (2013) were slightly greater for every airway generation except the trachea, despite the similarity in the body mass of the mice. This difference is likely due to the lung preparation procedure, which did not appear to conform to the pressure limitation standard for quantitative assessment of lung structure (Hsia et al., 2010) when the contrast agent was pumped into the mouse lungs. Comparison of our manual and CT‐derived airway measurements of the C57BL/6 in situ lung casts with those of Thiesse et al. (2005, 2010) on a branch‐by‐branch basis is difficult because of the differences between the binary number scheme used in this study, differences in specimen preparation, morphometry techniques, and their tracheobronchial airway nomenclature as well as the body mass differences in the two C57BL/6 mice they used. Thiesse et al. (2005) used excised lungs that were fix prior to CT‐derived measurements so differences in airway morphometry measurements could have occurred due lack of a chest cavity and tissue compliance. Thiesse et al. (2010) used respiratory gated CT‐derived measurements in live mice, however their airway dimensions were only obtained at midpoint of the airway length. The body mass of the two mice used by Thiesse et al. (2005, 2010) bracket (one lower, at 23.01 g, and one higher, at 29.5 g) the body mass of the C57LB/6 mice used in the present study (Table 1). Considering the differences in tissue preparation for their early work (Thiesse et al., 2005) and their morphometry techniques in their later work (measurements only taken at the middle of airway length), our manual and CT‐derived airway measurements are consistent (Figure 8) with those of Thiesse et al. (2005, 2010). Our manual and CT‐derived airway measurements are also consistent (Figure 8) with those Bauer et al. (2020) for Balb/c and C57BL6 mice.

The manual and automated airway morphometry data of the upper tracheobronchial airways of the Balb/c mice differed significantly from those of the C57BL/6 and Apoe^−/−^ mice, which highlights the importance of tracheobronchial anatomy for estimation of inhalation dosimetry. The similarities between the C57BL/6 mice and Apoe^−/−^ mice are not surprising, because the Apoe^−/−^ mouse was derived from the C57BL/6 mouse. In aerosol dosimetry programs differences airway diameter can lead to twice the difference in predicted particle deposition efficiency for micron‐sized aerosols (Phalen, Schum, & Oldham, 1990). By providing inclination to gravity of each airway, the automated airway morphometry is well suited to use in aerosol dosimetry models and computational fluid dynamic techniques that predict aerosol deposition in the respiratory tract. All of the micro‐CT scans obtained in this study, along with the resulting automated airway morphometry data, can be accessed on the INTERVALS website (https://www.intervals.science/).

## Supporting information

**FIGURE S1** Comparison of airway diameter (top), length (middle), and branch angle (bottom) obtained from manual airway morphometry measurements of two in‐situ lung Casts, #1 and #2 (Balb/c), reported by Islam, Oldham, and Wexler (2017) and averaged over each generation. Automated measurements of the present study (open circles) and those reported by Islam et al. (2017) (open boxes) for the first six tracheobronchial airway generations

**FIGURE S2** Average airway generation dimensions for airway length (top right), diameter (top right), branch angle (bottom left), and angle to gravity (bottom right) for AJ, C57BL6 and ApoE^−/−^ relative to the Balb/c strain

**TABLE S1** Quantitative comparison of the automated airway morphometry on an average generation basis for airway length among the four mouse strains. Generation in column 1, average measurement relative to Balb/c strain in columns 2–4, *p* value from ANOVA test in column 5, *p* values from pairwise *t* tests relative to Balb/c strain in column 6–8

**TABLE S2** Result of *t* test (*p* value) for the comparison of the automated airway morphometry on an average generation basis for airway diameter (D), length (L), branch angle (A), and angle to gravity (Ag) between adult male and female C57BL/6 mice (Cast 4 vs. Cast 7 and Cast 5 vs. Cast 6)

**DATA S1**: Supporting information.

## APPENDIX A.

### Micro‐CT image analysis workflow

Detailed workflow used to extract airway centerlines from the micro‐CT images using the Synopsys Simpleware software.

- MicroCT image segmentation:
  - Load DICOM images.
  - Set grayscale threshold: if cast is well filled, setting a slightly under resolved threshold will facilitate the subsequent opening operations.
  - Extract mask: keep only the larger island to maintain only the biggest connected volume.
  - Apply an open filter (about 2 pixel).
  - Flood fill of the main volume: with this operation you select only the connected volume that matches the selected target color.
  - Erode 1 pixel.
  - Open 1 pixel (see Figure A1).
  - Cavity Fill: this operation will fill (i.e., remove) any internal anomalies (e.g., bubbles, etc.) in the in‐situ lung cast.
  - Smoothing, 1.2 pixels.
  - Dilate 1 pixel.
  - Flood Fill.
  - Manually remove any remaining anomalies (e.g., bubbles on branch surface, or clumps of branches/alveola which may make the generation of the branches tree fail).
- Skelethonization:
  - Create centerlines.
  - Identify and resolve close loops or double branches.
  - Remove spurious nodes between only two branches.
  - Split each trifurcation with a sequence of bifurcations.
  - Inspect the centerlines and manually remove anomalies.
  - Rename branches and nodes based on Raabe et al. (1976) convention.
  - Smooth centerlines.
  - Extract statistics.
