# Supplementary material for: Use of micro‐CT to determine tracheobronchial airway geometries in three strains of mice used in inhalation toxicology as disease models
Source: Anat Rec (Hoboken). 2021 Mar 6;304(9):2050–67. doi: 10.1002/ar.24596 (PMC8451890; doi:10.1002/ar.24596)
Supplement: Supplementary file 3 — TABLE S1 Quantitative comparison of the automated airway morphometry on an average generation basis for airway length among the four mouse strains. Generation in column 1, average measurement relative to Balb/c strain in columns 2–4, p value from ANOVA test in column 5, p values from pairwise t tests relative to Balb/c strain in column 6–8 [file AR-304-2050-s005.docx]

**Table S1. Quantitative comparison of the automated airway morphometry on an average generation basis for airway length among the four mouse strains. Generation in column 1, average measurement relative to Balb/c strain in columns 2-4, p-value from ANOVA test in column 5, p-values from pairwise t-tests relative to Balb/c strain in column 6-8.**

| Length | | | | | | | |
| --- | --- | --- | --- | --- | --- | --- | --- |
|  | ratio L/L_balbc | | | ANOVA | T_Tests (p value) | | |
| Gen. | AJ | C57BL6 | ApoE | p | Balbc vs AJ | Balbc vs C57BL6 | Balbc vs ApoE |
| 1 | 0.95 | 0.78 | 1.19 | 0.686 | nan | 0.783 | 0.812 |
| 2 | 1.09 | 1.22 | 1.14 | 0.723 | 0.794 | 0.304 | 0.495 |
| 3 | 1.05 | 1.25 | 1.10 | 0.100 | 0.878 | 0.045 | 0.390 |
| 4 | 0.91 | 1.28 | 1.19 | 0.629 | 0.778 | 0.237 | 0.406 |
| 5 | 1.22 | 1.41 | 1.29 | 0.266 | 0.188 | 0.010 | 0.075 |
| 6 | 1.07 | 1.22 | 1.01 | 0.041 | 0.583 | 0.032 | 0.936 |
| 7 | 1.31 | 1.09 | 0.95 | 0.001 | 0.017 | 0.201 | 0.446 |
| 8 | 1.31 | 1.16 | 1.08 | 0.003 | 0.001 | 0.007 | 0.177 |

| Diameter | | | | | | | |
| --- | --- | --- | --- | --- | --- | --- | --- |
|  | ratio D/D_balbc | | | ANOVA | T_Tests (p value) | | |
| Gen. | AJ | C57BL6 | ApoE | p | Balbc vs AJ | Balbc vs C57BL6 | Balbc vs ApoE |
| 1 | 1.08 | 1.30 | 1.10 | 0.197 | nan | 0.120 | 0.483 |
| 2 | 1.11 | 1.54 | 1.41 | 0.021 | 0.646 | 0.001 | 0.010 |
| 3 | 1.17 | 1.60 | 1.66 | 0.090 | 0.525 | 0.007 | 0.007 |
| 4 | 1.23 | 1.50 | 1.42 | 0.208 | 0.351 | 0.008 | 0.040 |
| 5 | 1.04 | 1.54 | 1.37 | 0.023 | 0.812 | 0.001 | 0.018 |
| 6 | 0.99 | 1.31 | 1.30 | 0.065 | 0.938 | 0.011 | 0.017 |
| 7 | 1.01 | 1.24 | 1.32 | 0.022 | 0.926 | 0.007 | 0.002 |
| 8 | 0.95 | 1.13 | 1.10 | 0.204 | 0.639 | 0.058 | 0.187 |

| Branch Angle | | | | | | | |
| --- | --- | --- | --- | --- | --- | --- | --- |
|  | ratio A/A_balbc | | | ANOVA | T_Tests (p value) | | |
| Gen. | AJ | C57BL6 | ApoE | p | Balbc vs AJ | Balbc vs C57BL6 | Balbc vs ApoE |
| 1 | 0.00 | inf | inf | 0.272 | nan | 0.423 | 0.064 |
| 2 | 0.83 | 1.33 | 1.21 | 0.311 | 0.481 | 0.215 | 0.372 |
| 3 | 1.24 | 1.05 | 1.03 | 0.950 | 0.721 | 0.883 | 0.930 |
| 4 | 1.28 | 1.03 | 1.06 | 0.738 | 0.355 | 0.844 | 0.751 |
| 5 | 1.11 | 1.15 | 1.20 | 0.512 | 0.464 | 0.201 | 0.101 |
| 6 | 1.12 | 1.16 | 1.16 | 0.269 | 0.360 | 0.046 | 0.056 |
| 7 | 0.99 | 1.08 | 1.06 | 0.530 | 0.913 | 0.244 | 0.351 |
| 8 | 0.95 | 1.03 | 1.06 | 0.206 | 0.388 | 0.492 | 0.174 |

| Angle to Gravity | | | | | | | |
| --- | --- | --- | --- | --- | --- | --- | --- |
|  | ratio Ag/Ag_balbc | | | ANOVA | T_Tests (p value) | | |
| Gen. | AJ | C57BL6 | ApoE | p | Balbc vs AJ | Balbc vs C57BL6 | Balbc vs ApoE |
| 1 | 1.00 | 1.00 | 1.00 | 0.000 | . | 0.000 | 0.000 |
| 2 | 0.95 | 1.08 | 1.04 | 0.508 | 0.598 | 0.325 | 0.538 |
| 3 | 0.88 | 1.08 | 1.04 | 0.286 | 0.393 | 0.393 | 0.675 |
| 4 | 0.92 | 1.00 | 1.02 | 0.946 | 0.636 | 0.987 | 0.899 |
| 5 | 0.92 | 1.01 | 1.04 | 0.675 | 0.522 | 0.937 | 0.610 |
| 6 | 0.90 | 0.96 | 0.98 | 0.608 | 0.218 | 0.477 | 0.779 |
| 7 | 0.94 | 0.97 | 0.99 | 0.665 | 0.346 | 0.509 | 0.914 |
| 8 | 0.94 | 1.01 | 1.04 | 0.261 | 0.324 | 0.868 | 0.354 |
