# Supplementary material for: Use of micro‐CT to determine tracheobronchial airway geometries in three strains of mice used in inhalation toxicology as disease models
Source: Anat Rec (Hoboken). 2021 Mar 6;304(9):2050–67. doi: 10.1002/ar.24596 (PMC8451890; doi:10.1002/ar.24596)
Supplement: Supplementary file 4 — TABLE S2 Result of t test (p value) for the comparison of the automated airway morphometry on an average generation basis for airway diameter (D), length (L), branch angle (A), and angle to gravity (Ag) between adult male and female C57BL/6 mice (Cast 4 vs. Cast 7 and Cast 5 vs. Cast 6) [file AR-304-2050-s001.docx]

**Table S2. Result of t-test (p value) for the comparison of the automated airway morphometry on an average generation basis for airway diameter (D), length (L), branch angle (A) and angle to gravity (Ag) between adult male and female C57BL/6 mice (cast 4 vs. cast 7 and cast 5 vs. cast 6).**

|  | p value, T_test | | | | | | | |
| --- | --- | --- | --- | --- | --- | --- | --- | --- |
|  | cast #4 vs #7 | | | | cast #5 vs #6 | | | |
| Gen. | D | L | A | Ag | D | L | A | Ag |
| 1 | - | - | - | - | - | - | - | - |
| 2 | 0.82 | 0.86 | 0.94 | 0.96 | 0.56 | 0.87 | 0.14 | 0.24 |
| 3 | 0.77 | 0.69 | 0.74 | 0.86 | 0.61 | 0.35 | 0.63 | 0.42 |
| 4 | 0.62 | 0.87 | 0.97 | 0.75 | 0.29 | 0.97 | 0.81 | 0.70 |
| 5 | 0.98 | 0.88 | 0.94 | 0.50 | 0.11 | 0.75 | 0.38 | 0.56 |
| 6 | 0.69 | 0.06 | 0.95 | 0.45 | 0.05 | 0.22 | 0.18 | 0.83 |
| 7 | 0.65 | 0.01 | 0.70 | 0.03 | 0.22 | 0.39 | 0.15 | 0.60 |
| 8 | 0.50 | 0.11 | 0.25 | 0.16 | 0.36 | 0.14 | 0.01 | 0.70 |
